# Laparoscopic-Assisted Hysteroscopic Surgery for Endometrial Tumors Arising after Pelvic Radiation Therapy

**DOI:** 10.1155/2020/8837232

**Published:** 2020-12-08

**Authors:** Yuji Tanaka, Kimiko Hirata, Mariko Takahashi, Masashi Kobayashi

**Affiliations:** Department of Obstetrics and Gynecology, Kohka Public Hospital, Minakuchichomatsuo 1256, Koka, Shiga 528-0074, Japan

## Abstract

Uterine tumors detected after pelvic radiation therapy are rare. We report a case in which an endometrial tumor developed after pelvic radiation therapy for cervical cancer. The patient was a 70-year-old female with a history of pelvic radiotherapy for locally advanced cervical cancer. After 12 months of radiation therapy, magnetic resonance imaging showed tumors in the uterine cavity, and positron emission tomography-computed tomography showed no tumors, except uterine tumors. Since radiation therapy-induced cervical stenosis was conducted, endometrial examination could not be performed without anesthesia. As these tumors were detected after radiation therapy for uterine cancer, malignancy was considered. A laparoscopic-assisted transcervical hysteroscopic resection was performed for the diagnosis and treatment of uterine tumors after radiation therapy. This operative method was useful and enabled us to perform complete resection, observe the abdominal cavity, prepare for the possibility of secondary hysterectomy, and improve safety.

## 1. Introduction

Uterine tumors detected after radiation therapy for corpus cancer are rare. In such cases, malignant tumors should be considered. We report a case wherein an endometrial tumor was observed after radiation therapy for cervical cancer. The patient successfully underwent laparoscopic-assisted transcervical hysteroscopic resection.

## 2. Case Presentation

**Figure 1:**
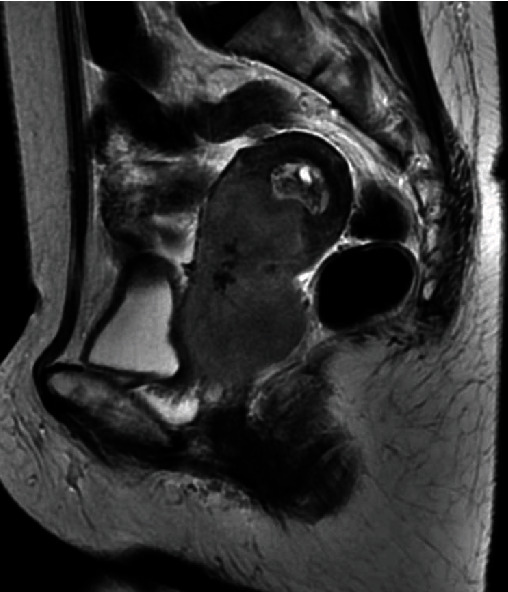
Magnetic resonance imaging (MRI) findings of primary disease. T2-weighted MRI showing locally advanced cervical cancer that extended into the endometrium.

A 70-year-old woman came to our hospital and presented with postmenopausal vaginal bleeding. The pathological diagnosis of cervical biopsy revealed adenocarcinoma. Computed tomography (CT) and magnetic resonance imaging (MRI) showed locally advanced cervical cancer that extended into the endometrium and pelvic sidewall (Figure 1), as well as bilateral hydronephrosis, but no other metastatic tumor was observed. The patient was diagnosed with cervical cancer stage IIIB. She underwent concurrent chemoradiotherapy. External beam radiation therapy at 50.4 Gy with conformal blocking against the whole pelvis, boost radiation therapy, and brachytherapy were performed. Platinum-based concurrent chemotherapy (cisplatin 40 mg/m^2^ weekly) was also performed. After radiation therapy, a complete response was considered.

**Figure 2:**
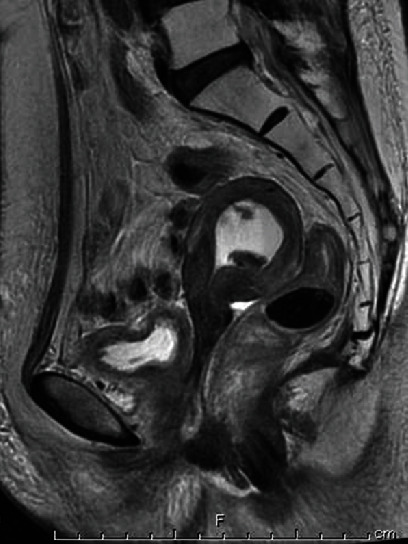
Magnetic resonance imaging (MRI) findings of endometrial tumors arising after pelvic radiation therapy. T2-weighted MRI showing markedly high-intensity liquid and two tumors in the uterine cavity (arrows).

After 12 months of concurrent chemoradiotherapy, ultrasonography showed tumors in the uterine cavity and pyometra. The serum CA-125 value was 41 U/ml. T2-weighted MRI showed markedly high-intensity liquid and tumors in the uterine cavity. Contrast-enhanced MRI showed two tumors in the uterine cavity with an enhancement effect (Figure 2). Positron emission tomography-CT showed no clear uptake in the whole body. The tumors' major axis growth was 8 mm to 15 mm in 3 months. Since radiation therapy-induced cervical stenosis was conducted, endometrial examination could not be performed without anesthesia.

**Figure 3:**
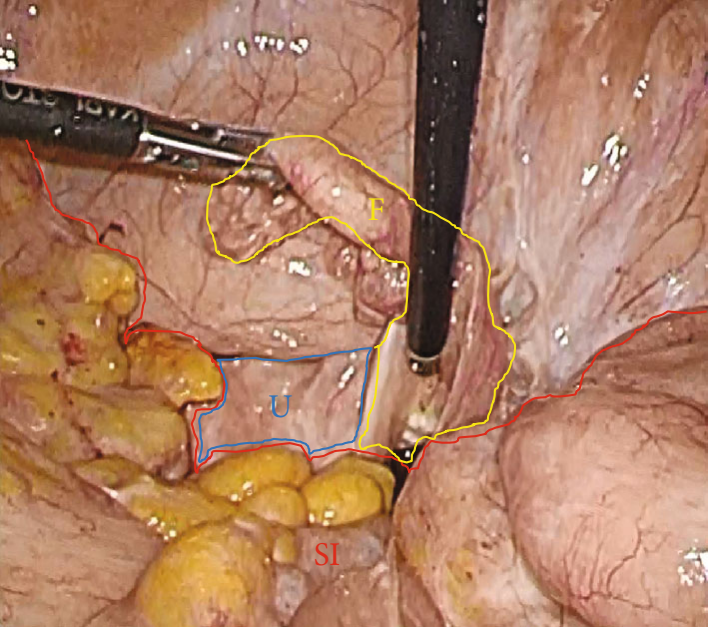
Laparoscopic findings. The uterus (U) was atrophied by radiation therapy and strongly adhered to the small intestine (SI); mobility was very poor. The fallopian tube (F) could be detected.

**Figure 4:**
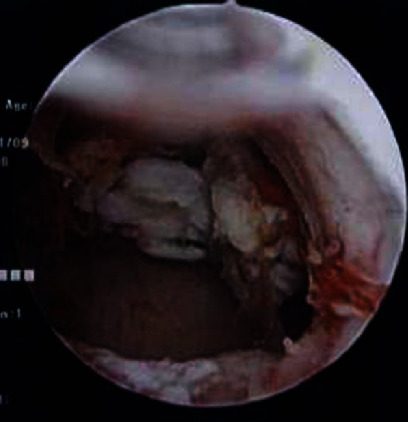
Hysteroscopic findings. Two tumors were detected in the uterine cavity.

**Figure 5:**
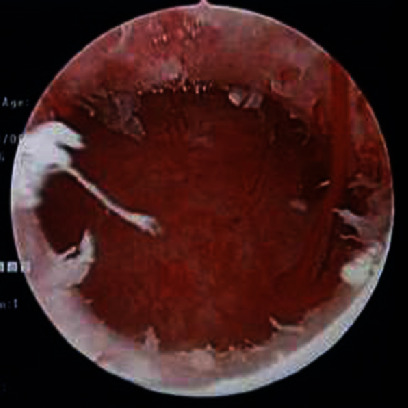
Hysteroscopic findings. At the end of the surgery, the uterine cavity was clear.

A laparoscopic-assisted transcervical hysteroscopic resection was performed for the diagnosis and treatment. At first, the abdominal cavity was observed laparoscopically (Figure 3). The mobility of the uterus was very poor due to its strong adhesion by radiation therapy. Bilateral fallopian tubes were sealed with a bipolar coagulator. Secondly, after watchful cervical dilation, the uterine cavity was observed hysteroscopically (Figure 4). An atrophic endometrium and two tumors were confirmed. The tumors were excised with an electric scalpel and discharged outside the uterus (Figure 5). The tumor stump and other parts of the endometrium were biopsied. The hysteroscopic surgery was laparoscopically monitored to prevent complications. Pathological diagnosis revealed endometrial polyps, which were stump-negative. At the 2-year follow-up postsurgery, there was no tumor recurrence.

The patient provided informed consent for the publication of the manuscript.

## 3. Discussion

In the case of “uterine tumor detected after radiation therapy for cervical cancer,” the differential diagnosis is expected to include recurrent tumors of primary cancer, radiation-induced malignant tumors, and endometrial polyps. Numerous studies have reported the development of recurrent uterine tumors or a second primary malignancy, such as sarcoma, after therapeutic pelvic irradiation [1–6]. Therefore, in such cases, measures assuming malignancy, specifically preparation for a secondary total hysterectomy, should be considered. Because the perioperative complication risk of hysterectomy after pelvic radiation therapy is high, complete tumor resection may be considered for these malignant tumors as a less invasive alternative treatment.

Radiation therapy can cause cervical stenosis, making endometrial examination difficult. In such cases, surgery under anesthesia may be required to obtain a pathological diagnosis.

In this case, we performed laparoscopic-assisted hysteroscopic surgery for uterine tumors detected after radiation therapy in uterine cancer for the diagnosis and treatment. We believe that this procedure has the following advantages: (1) A hysteroscopic approach allows complete resection of the tumor if a pathological diagnosis is conducted during surgery on the tumor stump. (2) The laparoscopic assistance approach was useful to assess the feasibility for secondary hysterectomy through the observation of the pelvic cavity. (3) Laparoscopic sealing of bilateral fallopian tubes might prevent the tumor cells from spreading into the peritoneal cavity, which is associated with hysteroscopic surgery through the fallopian tubes. (4) It can improve the safety of hysteroscopic surgery by the laparoscopic observation. As a result, a diagnosis of endometrial polyps was obtained in this case, and complete resection of the tumor was achieved. This procedure might be applied to a malignant tumor case with severe complications and difficult-to-perform highly invasive surgery.

In conclusion, the diagnosis and treatment of uterine tumors detected after radiation therapy for cervical cancer with cervical stenosis can be challenging. However, laparoscopically assisted hysteroscopic surgery could be of use for the diagnosis and treatment in such cases.

## Data Availability

The data that support the findings of this study are available from the corresponding author upon reasonable request.
